# Segmentation of brain magnetic resonance images based on multi-atlas likelihood fusion: testing using data with a broad range of anatomical and photometric profiles

**DOI:** 10.3389/fnins.2015.00061

**Published:** 2015-03-03

**Authors:** Xiaoying Tang, Deana Crocetti, Kwame Kutten, Can Ceritoglu, Marilyn S. Albert, Susumu Mori, Stewart H. Mostofsky, Michael I. Miller

**Affiliations:** ^1^Center for Imaging Science, Johns Hopkins UniversityBaltimore, MD, USA; ^2^Laboratory for Neurocognitive and Imaging Research, Kennedy Krieger InstituteBaltimore, MD, USA; ^3^Department of Biomedical Engineering, Johns Hopkins University School of MedicineBaltimore, MD, USA; ^4^Department of Neurology, Johns Hopkins University School of MedicineBaltimore, MD, USA; ^5^Johns Hopkins Alzheimer's Disease Research Center, Johns Hopkins University School of MedicineBaltimore, MD, USA; ^6^Russell H. Morgan Department of Radiology and Radiological Science, Johns Hopkins University School of MedicineBaltimore, MD, USA; ^7^F.M. Kirby Research Center for Functional Brain Imaging, Kennedy Krieger InstituteBaltimore, MD, USA; ^8^Department of Psychiatry, Johns Hopkins University School of MedicineBaltimore, MD, USA

**Keywords:** skull-stripping, automated brain segmentation, subcortical structures, ventricle, multi-atlas, likelihood-fusion

## Abstract

We propose a hierarchical pipeline for skull-stripping and segmentation of anatomical structures of interest from T1-weighted images of the human brain. The pipeline is constructed based on a two-level Bayesian parameter estimation algorithm called multi-atlas likelihood fusion (MALF). In MALF, estimation of the parameter of interest is performed via maximum a posteriori estimation using the expectation-maximization (EM) algorithm. The likelihoods of multiple atlases are fused in the E-step while the optimal estimator, a single maximizer of the fused likelihoods, is then obtained in the M-step. There are two stages in the proposed pipeline; first the input T1-weighted image is automatically skull-stripped via a fast MALF, then internal brain structures of interest are automatically extracted using a regular MALF. We assess the performance of each of the two modules in the pipeline based on two sets of images with markedly different anatomical and photometric contrasts; 3T MPRAGE scans of pediatric subjects with developmental disorders vs. 1.5T SPGR scans of elderly subjects with dementia. Evaluation is performed quantitatively using the Dice overlap as well as qualitatively via visual inspections. As a result, we demonstrate subject-level differences in the performance of the proposed pipeline, which may be accounted for by age, diagnosis, or the imaging parameters (particularly the field strength). For the subcortical and ventricular structures of the two datasets, the hierarchical pipeline is capable of producing automated segmentations with Dice overlaps ranging from 0.8 to 0.964 when compared with the gold standard. Comparisons with other representative segmentation algorithms are presented, relative to which the proposed hierarchical pipeline demonstrates comparative or superior accuracy.

## Introduction

To analyze how the pathology of a brain disorder affects specific anatomical structures and to understand the association between various cognitive functions and the anatomical phenotypes of specific brain structures, it is a necessity to first segment those brain structures of interest. To be feasible for large-scale neuroimaging studies, fully automated and highly accurate segmentation algorithms are in great demand. To this end, atlas-based segmentation algorithms have been widely explored (Miller et al., 1993; Collins and Evans, 1997; Haller et al., 1997; Hogan et al., 2000; Baillard et al., 2001; Crum et al., 2001; Carmichael et al., 2005; Heckemann et al., 2006; Haegelen et al., 2013). A brain atlas incorporates the information of numerous brain structures so as to guide the delineation of those structures in other to-be-segmented brain images. The simplest format is a pictorial representation of certain brain structures, guiding anatomists in defining the boundaries between neighboring structures. In computational algorithms, a brain atlas usually refers to a pair of images—the magnetic resonance (MR) image (e.g., T1-weighted image) and the corresponding anatomical structure definitions that are usually pre-defined via manual delineations by neuroanatomists. To better account for anatomical variability, it is natural to utilize the information of multiple atlases, which leads to a variety of multi-atlas based segmentation methods (Warfield et al., 2004; Aljabar et al., 2009; Langerak et al., 2010; Lotjonen et al., 2010; Asman and Landman, 2012; Tang et al., 2013; Wang and Yushkevich, 2013).

A crucial step in multi-atlas based segmentation is to register the MR image of each atlas to that of the to-be-segmented subject. To ensure accurate registration between two MR brain images, it usually requires a preliminary step to isolate the brain from other “non-brain” regions, a process which is referred to as “skull-stripping.” This is largely justified by the reliance of most registration algorithms upon the intensity information, as well as the geometric features, of the atlas and the subject MR images. The large variability of the “non-brain” regions, in terms of both intensity and geometry, often causes noise and makes the registration challenging. In addition to improving the registration accuracy between MR images, skull-stripping is also an important preliminary step in many other MR image processing procedures such as surface rendering and cortical flattening. A number of automated approaches to skull-stripping have been proposed and are widely used (Shattuck et al., 2001; Suri, 2001; Ségonne et al., 2004; Zhuang et al., 2006; Park and Lee, 2009). The accuracy and reliability of most automated skull-stripping approaches are affected by a range of factors, including the subject's age, diagnosis, resolution of the MR image, as well as the intensity bias presented in the MR image.

Skull-stripping and atlas-based segmentation are usually treated as two separate processes, given that they rely on different types of image processing techniques. Skull-stripping is primarily based on intensity thresholding, morphology, watershed transform, and hybrid methods while atlas-based segmentation focuses specifically on image registration.

We herein propose a hierarchical segmentation pipeline for unifying skull-stripping and structure extraction from T1-weighted images of the human brain in a Bayesian parameter estimation setting. This fully automated hierarchical pipeline is implemented in the MriCloud platform (www.mricloud.org). The pipeline is built on a segmentation label estimation algorithm called multi-atlas likelihood fusion (MALF) (Tang et al., 2013). MALF relies on the information of multiple atlases, each of which consists of an MR brain image and a pre-defined segmentation map. For any to-be-segmented subject image, we assume that every atlas image is a possible generator of it. Thus, in the estimation of the segmentation label, the choice of the deformable atlas has become a random variable. Integral to this estimation is identification of the optimal hidden diffeomorphism, acting on the background space of coordinates that effects the evolution with least energy from the randomly selected atlas image to the subject image. Maximum a posteriori estimation is employed in MALF, which is solved by iterating between fixing the locally optimized diffeomorphisms and obtaining the maximizing segmentation labels, then optimizing the local diffeomorphisms for the fixed segmentation in an expectation-maximization (EM) (Dempster et al., 1977) fashion.

In this paper, we first briefly describe the key idea of the MALF algorithm and then present our two-level hierarchical pipeline for skull-stripping and segmenting T1-weighted images that consists of two stages; skull-stripping of the input T1-weighted image followed by segmentation of the internal brain structures of interest. The performance of the proposed pipeline is evaluated based on two datasets that differ remarkably in their anatomical phenotypes and the photometric features of their input T1-weighted images. The first dataset comes from 30 pediatric subjects [a mixture of typically developing (TD) children, children with attention deficit hyperactivity disorder (ADHD) and autism spectrum disorder (ASD)], the T1-weighted images of which were obtained from a 3.0 Tesla (3.0T) Magnetization Prepared Rapid Gradient Recalled Echo (MPRAGE) imaging system. The second dataset is composed of 16 elderly subjects (a mixture of normal aging subjects, subjects with Alzheimer's disease (AD), mild cognitive impairment (MCI), and impairment but not MCI, the scans of which were obtained from 1.5 T coronal Spoiled Gradient Echo (SPGR) imaging systems.

One of the primary goals of this paper is to validate the broad range of applicability of the proposed pipeline, in terms of both skull-stripping and segmentation of subcortical and ventricular structures. We do this using two datasets with very different profiles in terms of age (pediatric vs. elderly), diagnosis (developmental disorders vs. dementia), and the imaging parameters (3.0T MPRAGE vs. 1.5T SPGR). The skull-stripping performance of the proposed pipeline is evaluated by comparing with two state-of-the-art skull-stripping methods for T1-weighted images [Hybrid Watershed Algorithm (HWA) (Ségonne et al., 2004) and Brain Extraction Tool (BET) (Smith, 2002)]. In evaluating the segmentation accuracy for subcortical and ventricular structures, we first compare the automated segmentation results obtained from the proposed pipeline with those from two widely used segmentation software packages [Freesurfer (Fischl et al., 2002) and FSL (Patenaude et al., 2011)] that are publicly available. In addition, to compare our pipeline with other multi-atlas based segmentation approaches, we assess the segmentation performance of the proposed pipeline relative to that of three representative label-fusion based segmentation algorithms [STAPLE (Warfield et al., 2004), Spatial STAPLE (Asman and Landman, 2012)] and a joint label-fusion approach (Wang et al., 2013) for which we use the term “ANTS+PICSL.” All methodology comparisons are performed based on the two aforementioned datasets.

## Materials and methods

### MALF

Assume that there exist *N* atlases, pairs of images {(*I_a_*, *W_a_*)}, where *I_a_* denotes the T1-weighted image of atlas *a* and *W_a_* denotes the paired segmentation label image. The *W_a_* is a map from the image domain Ω to a subset of the non-negative integers; *W_a_* (*x*) = 0 for voxel *x* ∈ Ω belonging to the unlabeled background, and *W_a_* (*x*) = *k*, *k* ∈ {1, 2, 3, …} for voxel *x* labeled as the *k*-th structure (such as the left caudate, the right putamen and so on). The segmentation label images of the atlases are usually pre-defined by neuroanatomists via manual delineations.

In MALF, the goal is to obtain the optimal estimator of the segmentation label *W* based on the observable image *I^D^* with optimality quantified by the segmentation accuracy when compared with the gold standard, the manual segmentations. We approach this problem via Bayesian estimation, which ensures a straightforward incorporation of information from multiple deformable atlases (*I_a_*, *W_a_*), *a* = *a_1_*, *a_2_*, …. We maximize the conditional probability of the parameter *W*, conditioned on the observed image *I^D^*, given by

(1) $$\hat{W}=\underset{W}{\arg\max}p(W|I^{D}).$$

The MAP estimator, defined in Equation (1), is equivalently obtained via

(2) $$\hat{W}=\underset{W}{\arg\max}p(I^{D},W),$$

where *p*(*I^D^*, *W*) is computed via a fusion of likelihoods from multiple deformable atlases, as

(3) $$p(I^{D},W)=\sum_{a}p(I^{D},W|A=a)\pi_{A}(a),$$

with π_*A*_(*a*) the prior probability that the observed image evolves from the specific atlas image *I_a_*, which is usually taken as the uniform distribution. Given a set of deformable atlas images {*I_a_*}, the possible starting points of the evolution process, the acquisition of the observable image *I^D^* can be mathematically formulated as *I^D^* = *I_A_* ° φ^−1^ + ε, where *I_A_* ∈ {*I_a_*}, φ ∈ 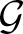 with 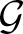 denoting the group of diffeomorphisms (bijective and smooth changes of coordinates on the background space with smooth inverses), and ε ~ Gaussian.

Equation (2) follows from the fact that *p*(*I^D^*, *W*) = *p*(*W*|*I^D^*)*p*(*I^D^*) and that *p*(*I^D^*) is constant with respect to the parameter *W*. Given a single atlas *a*, the likelihood model for inference is *p*(*I^D^*, *W*|*A* = *a*). With multiple atlases generating the observed image, the fusion of the likelihood functions yields the multi-modal mixture model with the prior averaging over models. This is the generative model with which we score each atlas and the essence of the MALF algorithm.

Given the explicit mathematical relationship *I^D^* = *I_A_* ° φ^−1^ + ε, *I_A_* ∈ {*I_a_*}, φ ∈ 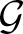, statistical estimation of the segmentation label *W* in the observed image *I^D^* can be made via a learning of the {(*I_a_*, *W_a_*)} and their mathematical connections to *I^D^*. Under this assumption that the observable data comes from a random deformation (randomness of the diffeomorphism φ) of a random atlas (randomness of the selected atlas *A*) with random noise, our probability model can be described as

(4) $$\begin{array}{l} p(W,I^{D},A=a,\varphi )=p(I^{D}|W,A=a,\varphi )p(W|\varphi ,A=a)\\ \;\pi (\varphi ,A=a), \end{array}$$

which comes from the conditional probability formula. The quantity *p*(*I^D^*|*W*, *A* = *a*, φ) is modeled as a pre-selected probability density function. In our case, we use a mixture of Gaussians, the parameters of which are estimated from the selected atlas *a*. The term *p*(*W*| φ, *A* = *a*) describes the prior information on the segmentation label *W*. More details about the theoretical foundations and algorithmic implementations of MALF can be found in Tang et al. (2013).

### Two-level hierarchical brain segmentation

**Figure 1:**
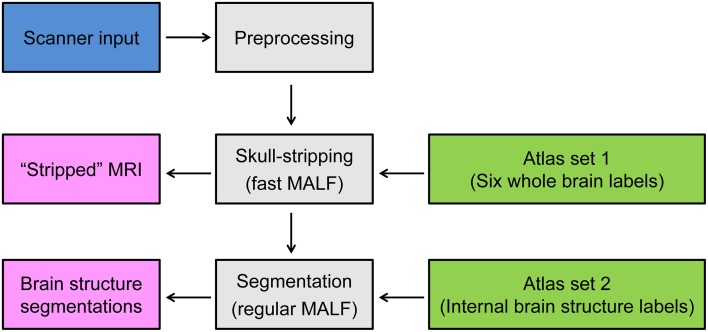
**Schematic of the proposed two-level hierarchical segmentation pipeline, consisting of three steps—preprocessing, first-level segmentation for skull-stripping, and second-level segmentation of subcortical and ventricular structures of interest**. Abbreviation: MALF, multi-atlas likelihood fusion.

The pipeline of the proposed two-level hierarchical brain segmentation is illustrated in Figure 1. After obtaining the T1-weighted image of the test subject from the scanner, we first re-orient the image and correct the inhomogeneity of the image intensity. We then perform the first-level segmentation to obtain the skull-stripped T1-weighted image of the subject. Following that, the second-level segmentation is performed to obtain the structures of interest for the input subject. Each step is detailed as follows:

1. *Preprocessing*: After the T1-weighted image of the subject is acquired from the scanner, its orientation is adjusted so that it matches the orientation of the atlas images. In our case, we use axial view as our standard orientation. We then correct for intensity-based inhomogeneities of the T1-weighted image for that subject using N3 (Sled et al., 1998). This inhomogeneity correction method is selected because of its applicability to T1-weighted images of the brain with skull and its superior performance (Arnold et al., 2001).
2. *Skull-stripping segmentation*: To obtain the skull-stripped T1-weighted image of the input subject, we estimate six global labels of the subject; the lateral ventricles (LV), the gray matter (GM) of the whole brain, the white matter (WM) of the whole brain, the cerebrospinal fluid (CSF) of the whole brain, the skull, and the background of the image. This is accomplished using T1-weighted images (for both the subject and the atlases) that have been down-sampled by a factor of two (e.g., from the original resolution of 1× 1× 1 mm to 2× 2× 2 mm). The timestep parameter is selected to be *T*=2 for the discretization in LDDMM image mapping (Beg et al., 2005) to approximate small deformations (Ceritoglu et al., 2013). The down-sampling of the images and the initial small deformations make the entire MALF procedure significantly faster. The four global labels, LV, GM, WM, and CSF, are grouped to create an initial brain mask, which is then morphologically post-processed to fill holes in the brain mask, remove grains, and smooth the rough boundary.
3. *Subcortical and ventricular segmentation*: After skull-stripping the T1-weighted image of the input subject, the structures of interest are obtained from a second iteration of the MALF algorithm. Here the stripped subject and atlas images are of the original image resolution (e.g., 1× 1× 1 mm). In this second iteration of MALF, to obtain the initial global optimal diffeomorphisms from LDDMM image mapping, we use the default timestep parameter *T* = 10. At the cost of being computationally intense, we are capable of achieving a more accurate segmentation for each internal brain structure of interest.

The aforementioned two-level hierarchical segmentation pipeline has been implemented in the MriCloud platform (www.mricloud.org). The computations were processed on the Gordon cluster of XSEDE (Towns et al., 2014). Each node on Gordon contains two 8-core 2.6 GHz Intel EM64T Xeon E5 (Sandy Bridge) processors and 64 GB of DDR3-1333 memory. The total segmentation time of one subject is around 70 min for an atlas set with 30 atlases using 4 nodes/64 cores.

### MRI dataset

Two datasets were used to evaluate the performance of the proposed pipeline in its two modules, skull-stripping and segmentation of subcortical and ventricular structures.

For the first dataset, high resolution T1-weighted 3D-volume MPRAGE coronal images covering the whole brain of 30 pediatric subjects were acquired from a Philips 3T “Achieva” MRI scanner (Best, the Netherlands) using an 8-channel head coil (*TR* = 7.99 ms, *TE* = 3.76 ms, Flip angle = 8°, voxel size = 1 mm isotropic). This dataset included 13 TD subjects (mean age: 10.42 years old; 5 males and 8 females), 6 male subjects with ASD (mean age: 9.74 years old) and 11 subjects diagnosed with ADHD (mean age: 10.2 years old; 4 males and 7 females). This study was approved by the Johns Hopkins Medical Institutional Review Board. Written consent was obtained from a parent/guardian and assent was obtained from the participating child.

The second dataset is a subset of scans collected in a longitudinal study focusing on dementia of the Alzheimer type [known as the BIOCARD study (Miller et al., 2013)]. T1-weighted images of 16 scans were obtained using a standard multi-modal protocol from a GE 1.5T scanner, with the scanning protocol being a coronal SPGR sequence (*TR* = 24, *TE* = 2, FOV = 256 × 256, thickness/gap = 2.0/0.0 mm, flip angle = 20, 124 slices). This dataset included 7 normal aging subjects (mean age: 60.09 years old; 4 males and 3 females), 2 subjects with MCI (mean age: 63.6 years old; 1 male and 1 female), 5 subjects with AD (mean age: 68.64 years old; 3 males and 2 females), and 2 subjects who are impaired but not MCI (mean age: 58.65 years old; 1 male and 1 female). This study was approved the Internal Review Board of the Johns Hopkins University School of Medicine.

### Creation of the atlas sets

The aforementioned two datasets served as two individual atlas sets. Leave-one-out (LOO) analysis was performed separately for each dataset; one subject was treated as the to-be-segmented subject and the remaining subjects of the same dataset served as the atlas set to segment the excluded subject. For each set, the orientation of each atlas image was adjusted to be axially oriented. All atlas images underwent inhomogeneity correction using the nonparametric non-uniform intensity normalization method N3 (Sled et al., 1998).

Since there are two levels of segmentation, there are correspondingly two sets of pre-defined segmentation labels for each atlas. The first set is used for the first-level segmentation (skull-stripping), consisting of the six global labels—the whole-brain GM, the whole-brain WM, the whole-brain CSF, the LV, the skull, and the background of the entire image. To create these labels for each atlas, its T1-weighted image was first manually skull-stripped by two raters (H.C. and X.T.) to separate the brain tissue from the non-brain regions. This manual skull-stripping procedure was performed using the software RoiEditor (http://www.MriStudio.org). Two labels, the skull and the background of the image, were then created by thresholding the intensity of voxels belonging to the non-brain regions in the T1-weighted image with the threshold value empirically selected. The lateral ventricles were manually traced by the same two raters (H.C. and X.T.). The other three labels, whole-brain GM/WM/CSF, were created by performing unified segmentation with respect to the manually skull-stripped T1-weighted image using a robust tissue segmentation algorithm (Ashburner and Friston, 2005) incorporated in the Statistical Parametric Mapping (SPM8) software. Given that the whole-brain GM/WM/CSF labels were obtained from an automated algorithm, we would expect some inaccuracy of these three labels. However, our priority is the final skull-stripping result, and we expect that extraction of the brain mask would not be significantly affected by an inaccuracy in obtaining the three GM/WM/CSF labels as long as the boundary separating the brain from the non-brain region is sufficiently accurate.

Regarding the second set of labels used for the purpose of the second-level segmentation, we treat the two datasets separately. For the first dataset, the label image of each atlas consists of 15 internal brain structures—left and right caudate, putamen, globus pallidus, thalamus, hippocampus, amygdala, local GM, local WM, and local CSF. The 6 basal ganglia structures (the bilateral caudate, putamen, and globus pallidus) were manually delineated by two anatomists (DC and KA) using the software MIPAV (Medical Image Processing, Analysis, and Visualization) (McAuliffe et al., 2001). The thalamus was manually delineated by two raters (XT and DC) using RoiEditor. The hippocampus and the amygdala were manually defined by two anatomists (EP and TB) using the software Seg3D (http://www.sci.utah.edu/cibc-software/seg3d.html). The other three labels—local GM/WM/CSF were obtained based on the approach described in (Tang et al., 2013). Briefly, we defined a cuboid region of interest (ROI) encompassing all 12 subcortical structures simultaneously in all atlases. Voxels inside this ROI but not belonging to any of the 12 manually delineated structures were automatically labeled as local GM/WM/CSF based on a local brain tissue segmentation algorithm (Priebe et al., 2006). The remaining voxels in the image domain but outside these 15 ROIs are unlabeled. The advantage of adding local surrounding tissue segmentation labels for the segmentation of subcortical structures has been demonstrated in Tang et al. (2013).

For the second dataset, each label image consists of a total of 13 internal brain structures, left and right putamen, globus pallidus, hippocampus, amygdala, lateral ventricle, local GM, local WM, and local CSF. The putamen and the globus pallidus in this dataset were manually delineated by two raters (NK and XT) using MIPAV. The amygdala and the hippocampus were manually traced by two anatomists (HT and TB) using Seg3D. The lateral ventricles were defined manually by two raters (HC and XT) using RoiEditor. The three local tissue labels were created in the same fashion as employed for the first dataset.

## Results

### Evaluation of the skull-stripping performance

#### Dataset 1

**Figure 2:**
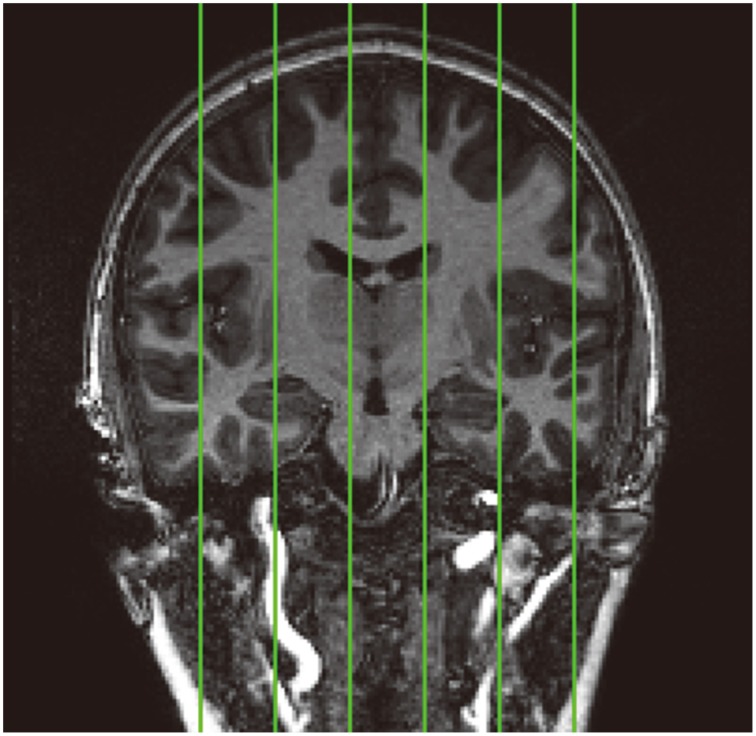
**Standard location of the six pre-selected sagittal slices (demonstrated on a coronal image) that have been manually skull stripped in the 30 pediatric images for validation analysis**. From left to right: slice1, slice2, slice3, slice4, slice5, and slice6.

In order to quantitatively assess the performance of the skull-stripping module in the proposed hierarchical pipeline, we compare the automated skull-stripping results with the manual delineations on six pre-selected sagittal slices (Figure 2) from the T1-weighted images of the 30 pediatric subjects. This comparison is quantified by the value of the Dice similarity coefficient (DSC) (Dice, 1945). Selection of the six sagittal slices follows the procedure described in (Fennema-Notestine et al., 2006).

**Figure 3:**
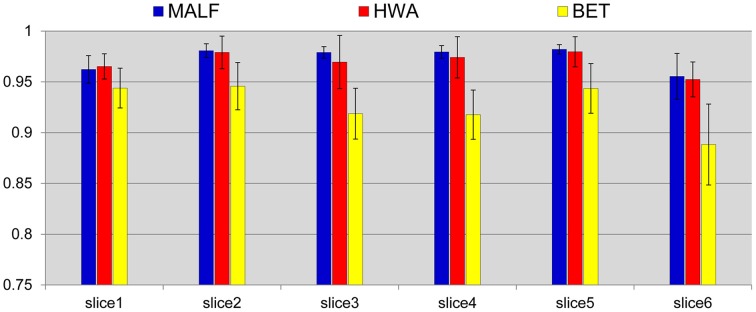
**Mean (std. error bar) Dice overlap values on the pre-selected slices of the skull-stripping results for the 30 pediatric subjects obtained from the three automated approaches—MALF (blue), HWA (red), and BET (yellow) compared with the corresponding manually stripped slices**.

We compare the skull-stripping results with those obtained from two of the most widely used skull-stripping methods—HWA (Ségonne et al., 2004) incorporated in the software Freesurfer (version 5.2.0) and BET (Smith, 2002) incorporated in the software FSL (version 5.0). Figure 3 shows the mean and the standard deviations of the DSC values for the three automated approaches, computed across all 30 subjects, for each of the six slices. According to paired Student's *t*-tests, the DSC values between the skull-stripping results from the proposed pipeline and those from manual delineations are, for all slices, statistically equivalent to the DSC values of the corresponding comparisons between HWA and manual delineations (*p* > 0.06). For each slice, the skull-stripping results from both the proposed pipeline and HWA are statistically superior to those from BET in terms of DSC (*p* < 1*e*^−5^).

**Figure 4:**
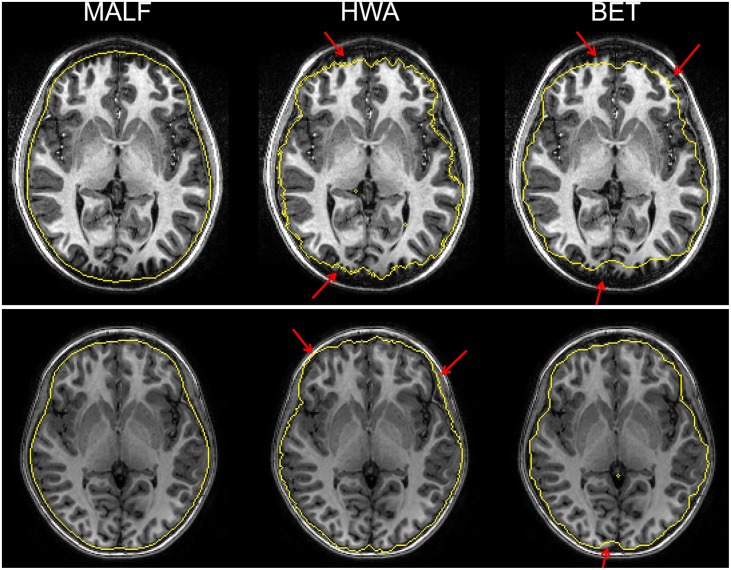
**The top and bottom panels show automated skull-stripping results of individual subjects of the pediatric dataset, different for each panel, superimposed on their corresponding T1-weighted image**. From left to right: results obtained from MALF, HWA, and BET. Regions pointed to with a red arrow indicate regions where inaccurate skull-stripping has occurred.

Upon visual inspection of the skull-stripping results on all slices for each image in this dataset, we observe that HWA sometimes misses cortical and cerebellar tissue. In two cases, it completely excludes the entire cerebellum region. HWA has also been found to act aggressively in regions close to the external dura, tending to include the entire external dura. The brain contour of the skull-stripped images from HWA is not smooth in a number of cases. Visual examination also suggests that BET consistently underestimates relevant brain tissue. In Figure 4, we demonstrate the skull-stripping results obtained from the three methods for two representative subjects. It is clear that for the subject displayed on the top panel of that figure, both HWA and BET miss some cortical brain tissue. For the subject on the bottom panel, HWA is inclined to include more non-brain tissue while BET misses some brain tissue. As illustrated by the yellow contour lines shown in Figure 4, the boundaries of the skull-stripped brain images from both HWA and BET are not as smooth as those from our hierarchical pipeline based on MALF.

#### Dataset 2

**Figure 5:**
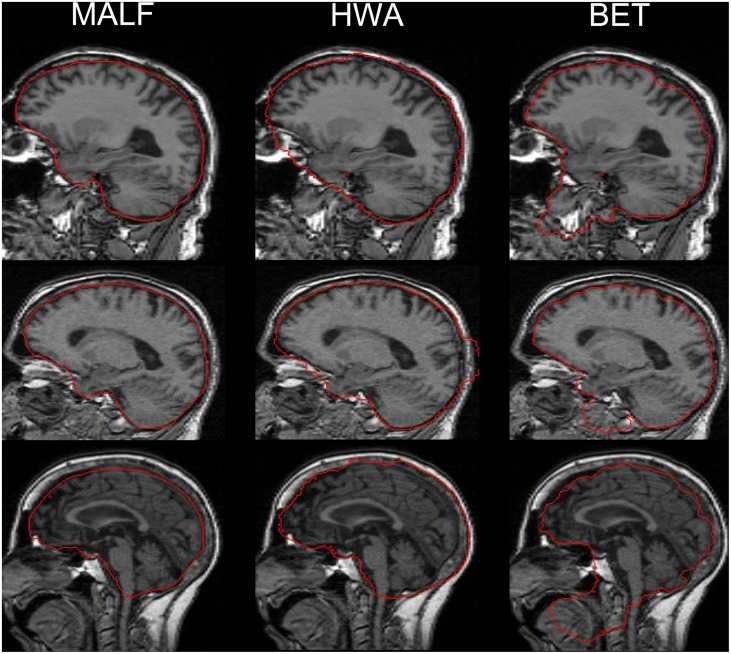
**A comparison of the three automated skull-stripping approaches (from left to right: results from MALF, HWA, and BET) based on three representative cases from the 16 elderly scans of the second dataset**. Red lines indicate the boundaries that separate the brain tissue defined by each method and the “non-brain” regions.

For the second dataset, the skull-stripping performance of the three automated methods (MALF, HWA, and BET) is compared qualitatively via a visual examination of the results for all 16 scans. Qualitative review of all of the individual results reveals that MALF is superior to both HWA and BET in at least two respects: Firstly, MALF performs an accurate separation between the brain tissue and the external CSF whereas HWA consistently includes the external CSF (the space between the brain tissue and the external dura) and sometimes bone marrow (the third example of HWA in Figure 5). Meanwhile, BET is typically over conservative, missing some portion of the cortical regions. Secondly, MALF does not mistakenly include non-brain regions such as the eye, the mouth, and the skull while HWA and BET both have various non-brain regions included in the “stripped” volumes. For HWA, regions adjacent to the eyes are often included (the first example of HWA in Figure 5) and sometimes parts of the skull are included (the second example of HWA in Figure 5). As for BET, large amounts of regions close to the mouth are included (all 3 examples of BET in Figure 5).

### Evaluation of the subcortical and ventricular segmentation accuracy

To evaluate the accuracy of the second module in our pipeline, segmenting subcortical and ventricular structures of interest, we compare the automated segmentations with the corresponding manual ones using the DSC value. For each dataset, segmentation accuracy of the proposed pipeline is first compared with that of Freesurfer (Fischl et al., 2002) and FSL (Patenaude et al., 2011), and then compared with three multi-atlas label-fusion based segmentation techniques—STAPLE (Warfield et al., 2004), Spatial STAPLE (Asman and Landman, 2012), and ANTS+PICSL (Wang et al., 2013). For both STAPLE and Spatial STAPLE, LDDMM was employed as the pairwise registration technique. For ANTS+PICSL, we followed the segmentation routine as suggested in Wang and Yushkevich (2013); the diffeomorphic registration module in ANTs, SyN (Avants et al., 2008), was used for the pairwise diffeomorphic registration followed by joint label fusion and corrective learning.

#### Comparison with freesurfer and FSL

**Figure 6:**
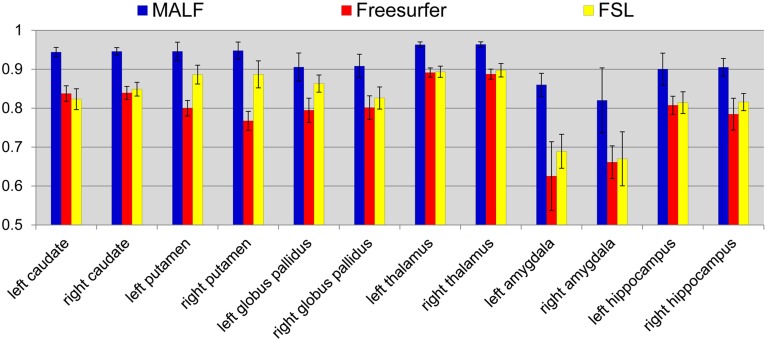
**The mean and standard deviations of the Dice overlap values for the 12 subcortical structures (left and right caudate, putamen, globus pallidus, thalamus, amygdala, and hippocampus) obtained from MALF (blue), Freesurfer (red), and FSL (yellow) relative to the manual delineations**. The mean values are computed across all of the 30 pediatric subjects.

**Figure 7:**
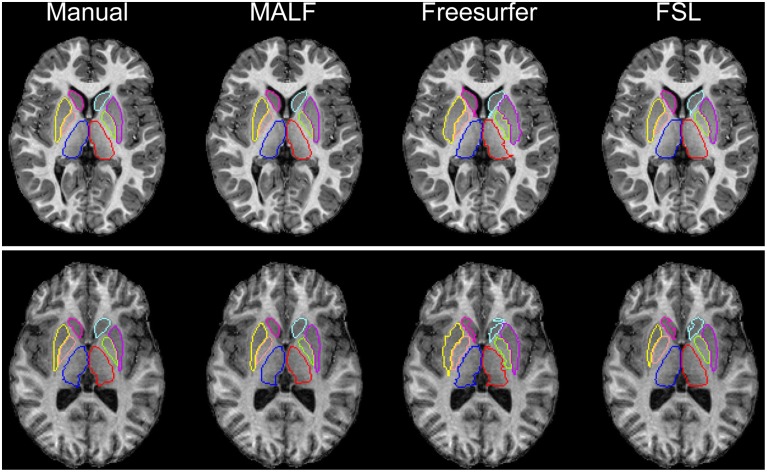
**The top and bottom panels show segmentation results of the six basal ganglia structures (left and right caudate, putamen, and globus pallidus) and the bilateral thalamus in an individual subject of the first dataset (30 pediatric subjects), different for each panel**. From left to right, segmentations are obtained from manual delineation, MALF, Freesurfer, and FSL.

**Figure 8:**
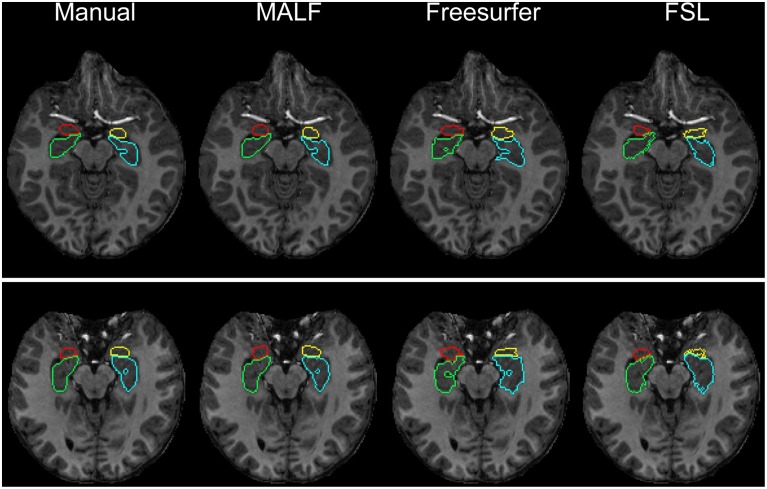
**The top and bottom panels show the segmentation results of the left amygdala (yellow), the right amygdala (red), the left hippocampus (blue), and the right hippocampus (green) in individual subjects from the pediatric dataset, different for each panel**. From left to right, segmentations are obtained from manual delineation, MALF, Freesurfer, and FSL.

***Dataset 1***. For the first dataset, the mean and the standard deviations of the DSC values, for all 12 subcortical structures produced by MALF, FreeSurfer, and FSL, are shown in Figure 6. Two-sampled Student's *t*-tests reveal that MALF is statistically significantly better than both Freesurfer and FSL in segmenting each of those 12 structures (*p* < 1*e*^−5^). Comparing Freesurfer and FSL, FSL performs better than Freesurfer in segmenting the right caudate, the bilateral putamen, the bilateral globus pallidus, the right thalamus, the left amygdala, and the right hippocampus (*p* < 5*e*^−3^), as evaluated by the DSC value. In Figure 7, we illustrate two examples of the basal ganglia and the thalamus segmentations, while Figure 8 gives examples for the amygdala and the hippocampus, from manual delineations as well as from the three automated approaches. The structure definitions are superimposed on the corresponding T1-weighted images.

**Figure 9:**
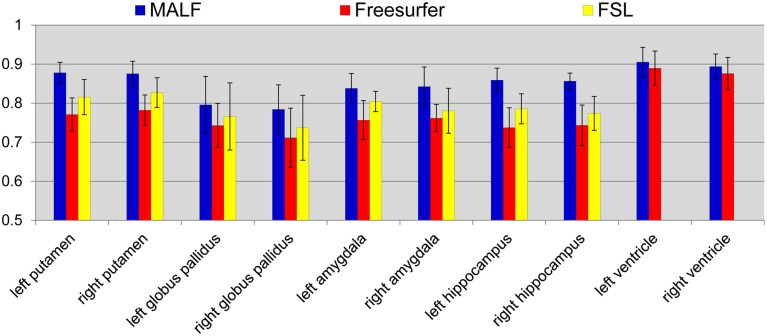
**The mean DSC values and the standard deviations of the 10 structures of interest (left and right putamen, globus pallidus, amygdala, hippocampus, and lateral ventricle) in the second dataset**. The values are computed from the automated segmentations of MALF (blue), Freesurfer (red), and FSL (yellow) relative to the corresponding manual ones.

**Figure 10:**
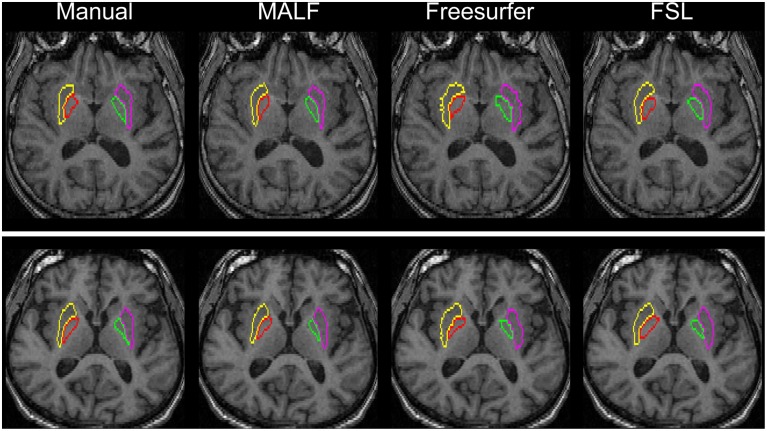
**Demonstration of the bilateral putamen and globus pallidus definition in two representative subjects from the second dataset (16 elderly scans)**. From left to right: results obtained from manual, MALF, Freesurfer, and FSL.

**Figure 11:**
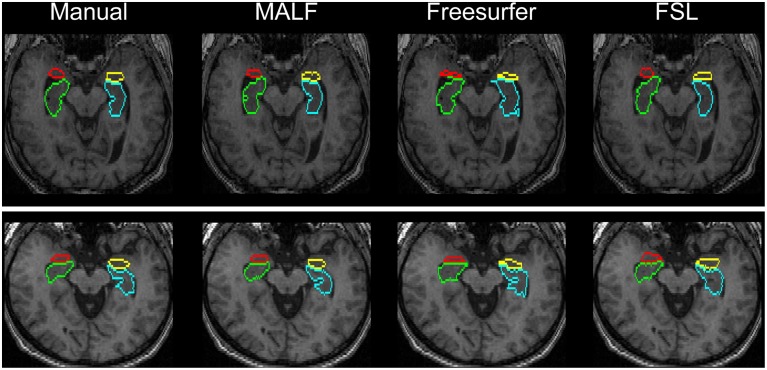
**Visual comparisons of the segmentations of the hippocampus and the amygdala in two representative subjects from the second dataset (16 elderly scans)**. The segmentations are obtained from manual delineation, MALF, Freesurfer, and FSL.

***Dataset 2***. For the 16 elderly scans from the second dataset, quantitative comparisons of MALF, Freesurfer, and FSL, in terms of the mean DSC values for each of the 10 structures of interest, are demonstrated in Figure 9, with the standard deviations plotted as the error bars. Paired Student's *t*-tests reveal that MALF is statistically significantly better than Freesurfer in segmenting the bilateral ventricles (left: *p* = 0.036, right: *p* = 0.042). Furthermore, MALF is statistically significantly superior to both Freesurfer and FSL in segmenting the other 8 structures with the exception of the right globus pallidus (left amygdala: *p* < 0.005, right amygdala: *p* < 0.002, left hippocampus: *p* < 2.3*e*^−6^, right hippocampus: *p* < 6.8*e*^−8^, left putamen: *p* < 1.5*e*^−8^, right putamen: *p* < 3*e*^−8^, left globus pallidus: *p* < 0.05, right globus pallidus: *p* > 0.05). Qualitative comparisons of the three methods in segmenting the putamen and the globus pallidus from images in this dataset are illustrated in Figure 10 while Figure 11 gives comparisons of the amygdala and the hippocampus.

#### Comparison with multi-atlas label-fusion based segmentation algorithms

***Dataset 1***. Table 1 tabulates the mean and the standard deviations of the DSC values, computed across the 30 pediatric subjects in the first dataset, from MALF and the three selected multi-atlas label-fusion based segmentation algorithms—STAPLE, Spatial STAPLE, and ANTS+PICSL. Paired Student's *t*-tests reveal that MALF provides superior segmentation performance relative to STAPLE for each of the 12 subcortical structures. Comparing MALF and Spatial STAPLE, MALF is statistically significantly better than Spatial STAPLE in segmenting the caudate, the putamen, the globus pallidus, and the hippocampus, all in both hemispheres. MALF and ANTS+PICSL are statistically equivalent in segmenting a majority of the 12 subcortical structures with the exceptions that MALF outperforms ANTS+PICSL in segmenting the bilateral amygdala while ANTS+PICSL is superior to MALF in segmenting the left globus pallidus.

**Table 1:** **The DSC values between the manual and automated segmentations, averaged across the 30 pediatric subjects, from MALF, ANTS+PICSL, Spatial STAPLE, and STAPLE**.

	**MALF**	**ANTS+PICSL**	**Spatial STAPLE**	**STAPLE**
Left caudate	**0.944 ± 0.012**	**0.944 ± 0.014**	0.915 ± 0.019	0.871 ± 0.033
Right caudate	**0.946 ± 0.009**	**0.946 ± 0.009**	0.914 ± 0.018	0.869 ± 0.035
Left putamen	**0.946 ± 0.023**	**0.949 ± 0.010**	0.931 ± 0.024	0.908 ± 0.026
Right putamen	**0.948 ± 0.022**	**0.949 ± 0.010**	0.933 ± 0.021	0.912 ± 0.024
Left globus pallidus	0.906 ± 0.036	**0.920 ± 0.016**	0.891 ± 0.032	0.864 ± 0.035
Right globus pallidus	**0.908 ± 0.031**	**0.917 ± 0.014**	0.886 ± 0.029	0.861 ± 0.034
Left thalamus	**0.963 ± 0.007**	**0.958 ± 0.015**	**0.967 ± 0.007**	0.949 ± 0.014
Right thalamus	**0.964 ± 0.006**	**0.958 ± 0.016**	**0.966 ± 0.006**	0.947 ± 0.011
Left amygdala	**0.861 ± 0.029**	0.829 ± 0.039	**0.861 ± 0.026**	0.772 ± 0.053
Right amygdala	**0.821 ± 0.083**	0.793 ± 0.079	**0.815 ± 0.078**	0.745 ± 0.105
Left hippocampus	**0.901 ± 0.041**	**0.895 ± 0.052**	0.878 ± 0.054	0.833 ± 0.059
Right hippocampus	**0.905 ± 0.023**	**0.905 ± 0.018**	0.888 ± 0.032	0.847 ± 0.041

*Bold typesetting indicates that the Dice overlap obtained from the corresponding method is statistically significant in being greater than that of other methods (*p* < 0.05)*.

***Dataset 2***. For the 16 elderly scans in the second dataset, we list the DSC values' mean and standard deviations, calculated from the four automated approaches (MALF, Spatial STAPLE, STAPLE, and ANTS+PICSL), in Table 2. According to the results from paired Student's *t*-tests, MALF is statistically significantly superior to STAPLE in segmenting all of the 10 structures of interest from this dataset. Compared with Spatial STAPLE, MALF produces significantly more accurate automated segmentations for the bilateral putamen, globus pallidus, amygdala, hippocampus, and the right lateral ventricle, while being statistically comparable in segmenting the left lateral ventricle. Comparing MALF and ANTS+PICSL, they are statistically equivalent in segmenting the bilateral globus pallidus, the bilateral hippocampus, and the left lateral ventricle. MALF is statistically superior to ANTS+PICSL in segmenting the right lateral ventricle whereas, for this dataset, ANTS+PICSL performs a better segmentation of the putamen and the amygdala in both hemispheres.

**Table 2:** **The DSC values between the manual and the automated segmentation volumes, averaged across the 16 elderly scans from the second dataset, the automated segmentations of which are obtained from MALF, ANTS+PICSL, Spatial STAPLE, and STAPLE**.

	**MALF**	**ANTS+PICSL**	**Spatial STAPLE**	**STAPLE**
Left putamen	0.878 ± 0.026	**0.898 ± 0.029**	0.861 ± 0.036	0.817 ± 0.047
Right putamen	0.875 ± 0.032	**0.896 ± 0.031**	0.851 ± 0.027	0.801 ± 0.042
Left globus pallidus	**0.796 ± 0.073**	**0.803 ± 0.056**	0.751 ± 0.085	0.701 ± 0.086
Right globus pallidus	**0.784 ± 0.063**	**0.789 ± 0.046**	0.743 ± 0.062	0.687 ± 0.072
Left amygdala	0.838 ± 0.038	**0.861 ± 0.033**	0.822 ± 0.043	0.827 ± 0.041
Right amygdala	0.843 ± 0.049	**0.862 ± 0.033**	0.829 ± 0.041	0.835 ± 0.031
Left hippocampus	**0.859 ± 0.031**	**0.849 ± 0.056**	0.823 ± 0.051	0.782 ± 0.074
Right hippocampus	**0.856 ± 0.021**	**0.848 ± 0.048**	0.831 ± 0.033	0.789 ± 0.056
Left ventricle	**0.912 ± 0.024**	**0.896 ± 0.076**	**0.891 ± 0.023**	0.867 ± 0.052
Right ventricle	**0.924 ± 0.018**	0.885 ± 0.073	0.874 ± 0.029	0.856 ± 0.049

*Bold typesetting indicates that the Dice overlap obtained from the corresponding method is statistically significant in being greater than that of other methods (*p* < 0.05)*.

## Discussion

In this paper, we have proposed a fully automated skull-stripping and segmentation pipeline for T1-weighted images based on two levels of MALF (Tang et al., 2013); the first level is a fast version of MALF used for skull-stripping with low computational cost and the second level is a regular version of MALF employed in pursuit of high segmentation accuracy. There are three steps in the whole pipeline: (1) pre-processing, (2) isolating the brain tissue from extracranial regions (skull-stripping), and (3) extracting the brain structures of interest from the “stripped” T1-weighted images.

The performance of the proposed hierarchical pipeline was evaluated based on two sets of brain images with very different profiles in terms of both anatomical phenotype (due to age and differences in diagnosis) and photometric profile (due to differences in the imaging parameters). The first dataset consisted of 30 pediatric subjects, a mixture of TD children, children with ADHD and ASD. T1-weighted images of this dataset were obtained from a 3T MPRAGE imaging system. The second dataset consisted of 16 elderly subjects, a mixture of normal aging, MCI, AD, as well as impaired but not MCI subjects. Images of the second dataset were obtained from a 1.5T SPGR imaging system. To evaluate the skull-stripping and segmentation accuracy of the proposed pipeline, we performed leave-one-out experiments on each dataset; one subject was treated as the to-be-processed target while the remaining subjects of the same dataset served as the multiple deformable atlases.

The skull-stripping performance of the hierarchical pipeline was shown to be comparable with that of the manual approach (Figure 3). Relative to two of the most popular skull-stripping algorithms (HWA and BET) for T1-weighted images, our approach provides superior performance for both datasets. As shown in Figures 4, 5, the proposed pipeline automatically produced highly precise skull-stripped brain images with noticeably smooth boundaries. A primary limitation that we have noticed of BET is that it is particularly inclined to underestimate relevant brain tissue around cortical regions (Figures 4, 5) while including a large amount of non-brain regions around the mouth (Figure 5) when using its default parameter setting. An investigation of the parameter settings may relieve this issue, an aspect that we did not pursue. The primary advantage of BET is that it also works for the skull-stripping of T2- and proton-density-weighted images whereas HWA and the proposed method only apply to T1-weighted images. As for HWA, it sometimes includes non-brain matter around the eyes or, conversely, excludes relevant brain tissue altogether (such as the superior frontal gyrus). In some extreme cases, HWA excludes the cerebellum completely. The superiority of the proposed pipeline in skull-stripping is more obviously established when applied to more difficult cases such as images in the second dataset. As shown in Figure 5, both HWA and BET are likely to commit more skull-stripping mistakes when applied to the elderly scans from the second dataset, while our pipeline excels. This observation agrees with the comprehensive analysis results in Fennema-Notestine et al. (2006), in which the T1-weighted images of subjects with AD especially those obtained from 1.5T scanners (possibly because of relatively poor image contrasts) were found to be the most difficult cases for typical skull-stripping algorithms. The success of the proposed pipeline in skull-stripping 1.5T scans diagnosed with AD and MCI is indicative of a wider applicability.

For the second module of our pipeline, i.e., segmentation of subcortical and ventricular structures, the proposed hierarchical pipeline was shown to be capable of creating precise automated segmentations for both datasets, demonstrating statistically significantly higher segmentation accuracy than both FreeSurfer and FSL (Figures 6–11). With that being said, the gap in performance may have been associated with a difference in terms of the manual delineation protocols for the training datasets; FreeSurfer and FSL both have their own training datasets, for which the definitions of subcortical and ventricular structures may vary between them, and may be different from the ones delineated in our atlases. Compared with three label-fusion based segmentation techniques—STAPLE, Spatial STAPLE, and ANTS+PICSL (Tables 1, 2)—our pipeline is superior (compared to STAPLE and Spatial STAPLE) or comparable (with respect to ANTS+PICSL) in terms of segmentation accuracy. Comparing the segmentation results across the two contrasting datasets, we find that the proposed pipeline achieves higher accuracy in segmenting the pediatric data, which came from a scanner with higher-field strength magnets (3T vs. 1.5T), than the elderly data that are normal aging or of a diagnostic variety ranging toward dementia of the Alzheimer type. This clearly suggests that the segmentation performance of the proposed pipeline is affected by the underlying dataset. These influential effects may be related with the test subjects' age, cognitive status, diagnosis, or the imaging parameters (particularly the field strength) that determine the contrast profile of the T1-weighted images. Additional data from a range of subjects of different ages and diagnostic categories, scanned at different field strengths would be required to determine which of these factors is contributing to the observed differences in the performance of this segmentation pipeline.

One aspect that is worthy of note is the fixing of certain parameters, independently of the target dataset. Usually, for a specific pipeline, parameters are tuned to best suit the current study. In the proposed pipeline, most parameters are estimated automatically using maximum-likelihood estimation, examples being the means and variances of the Gaussian mixture models (Tang et al., 2014). For parameters that need to be pre-assigned such as the LDDMM algorithm's timestep parameter or its ratio between the two terms of the energy function (Beg et al., 2005), they are selected based on prior testing on a large sample of datasets (much larger, in total, than the dataset used in the evaluation of this study). Instead of only pursuing the best performance in terms of accuracy, those fixed parameters in our pipeline were chosen to ensure pipeline stability as well as good performance.

To summarize, we proposed and validated a fully automated segmentation pipeline, built on a two-level multi-atlas likelihood fusion, for pediatric as well as elderly T1-weighted images. We illustrate the capability of our skull-stripping and segmentation pipeline in creating highly reliable and accurate skull-stripped images as well as segmentations of subcortical and ventricular structures from subjects with varying anatomical and photometric phenotype, demonstrating a wide applicability of the proposed pipeline. Each of the two modules, skull-stripping and brain structure segmentation, in the proposed pipeline is of great importance to a variety of medical image processing and clinical applications.

### Conflict of interest statement

The authors declare that the research was conducted in the absence of any commercial or financial relationships that could be construed as a potential conflict of interest.
